# Multidisciplinary consensus on screening for, diagnosis and management of fetal growth restriction in the Netherlands

**DOI:** 10.1186/s12884-017-1513-3

**Published:** 2017-10-16

**Authors:** Viki Verfaille, Ank de Jonge, Lidwine Mokkink, Myrte Westerneng, Henriëtte van der Horst, Petra Jellema, Arie Franx, IRIS study group, Joke Bais, Gouke J. Bonsel, Judith E. Bosmans, Jeroen van Dillen, Noortje T. L. van Duijnhoven, William A. Grobman, Henk Groen, Chantal W. P. M. Hukkelhoven, Trudy Klomp, Marjolein Kok, Marlou L. de Kroon, Maya Kruijt, Anneke Kwee, Sabina Ledda, Harry N. Lafeber, Jan M. van Lith, Ben Willem Mol, Bert Molewijk, Marianne Nieuwenhuijze, Guid Oei, Cees Oudejans, K. Marieke Paarlberg, Eva Pajkrt, Aris T. Papageorghiou, Uma M. Reddy, Paul A. O. M. De Reu, Marlies Rijnders, Alieke de Roon-Immerzeel, Connie Scheele, Sicco A. Scherjon, Rosalinde Snijders, Pim W. Teunissen, Hanneke W. Torij, Jos Twisk, Kristel C. Zeeman, Jun Zhang

**Affiliations:** 1 Midwifery Science, AVAG, Amsterdam Public Health research institute, VU University Medical Center, Van der Boechorststraat 7, 1081 BT Amsterdam, the Netherlands; 2 Department of Epidemiology and Biostatistics and Amsterdam Public Health research institute, VU University Medical Center, Van der Boechorststraat 7, 1081 BT Amsterdam, the Netherlands; 3 Department of General Practice, Amsterdam Public Health research institute, VU University Medical Center, Van der Boechorststraat 7, 1081 BT Amsterdam, the Netherlands; 4 Department of Gynecology, Utrecht University Medical Centre, Heidelberglaan 100, 3584 CX Utrecht, the Netherlands

**Keywords:** Intrauterine growth restriction, fetal growth restriction, Delphi technique, Practice guideline, Prenatal ultrasonography, Collaboration, Uniform approach

## Abstract

**Background:**

Screening for, diagnosis and management of intrauterine growth restriction (IUGR) is often performed in multidisciplinary collaboration. However, variation in screening methods, diagnosis and management of IUGR may lead to confusion. In the Netherlands two monodisciplinary guidelines on IUGR do not fully align. To facilitate effective collaboration between different professionals in perinatal care, we undertook a Delphi study with uniform recommendations as our primary result, focusing on issues that are not aligned or for which specifications are lacking in the current guidelines.

**Methods:**

We conducted a Delphi study in three rounds. A purposively sampled selection of 56 panellists participated: 27 representing midwife-led care and 29 obstetrician-led care. Consensus was defined as agreement between the professional groups on the same answer and among at least 70% of the panellists within groups.

**Results:**

Per round 51 or 52 (91% - 93%) panellists responded. This has led to consensus on 27 issues, leading to four consensus based recommendations on screening for IUGR in midwife-led care and eight consensus based recommendations on diagnosis and eight on management in obstetrician-led care. The multidisciplinary project group decided on four additional recommendations as no consensus was reached by the panel. No recommendations could be made about induction of labour versus expectant monitoring, nor about the choice for a primary caesarean section.

**Conclusions:**

We reached consensus on recommendations for care for IUGR within a multidisciplinary panel. These will be implemented in a study on the effectiveness and cost-effectiveness of routine third trimester ultrasound for monitoring fetal growth. Research is needed to evaluate the effects of implementation of these recommendations on perinatal outcomes.

**Trial registration:**

NTR4367.

## Background

Infants with intrauterine growth restriction (IUGR) are at increased risk for perinatal morbidity and mortality [1–4]. Therefore screening for, diagnosis and management of IUGR are important assignments for all caregivers in perinatal care [5–7].

IUGR is defined as the failure to achieve full fetal growth potential. Abdominal palpation or serial fundal height (SFH) measurements are primarily used in clinical practice to assess fetal growth. Additional diagnostic testing by ultrasound biometry is done if indicated, based on relevant history, pregnancy complications or clinical suggestion of IUGR based on abdominal palpation or SFH measurements [8, 9]. An estimated fetal weight (EFW) below the 10th centile of a population curve is most commonly used in literature and guidelines as a proxy for IUGR [10–14]. If IUGR is suspected, additional tests such as Doppler velocimetry can show redistribution patterns of blood flow, suggestive for the fetal adaptive response to suboptimal conditions, either caused by asphyxia or maternal malnutrition [15–19].

Perinatal care for IUGR requires multidisciplinary collaboration, as pregnant women may transit from low- to high-risk care during pregnancy. Consequently uniform multidisciplinary definitions and guidelines are required to reduce inconsistencies in the clinical management of IUGR, a challenge that has been recognised internationally [20–23]. In the Netherlands the guideline of the Royal Dutch Organisation of Midwives (KNOV) focuses on screening in the low-risk population, whereas the guideline of the Dutch Society of Obstetrics and Gynaecology (NVOG) focuses on diagnostics and management when IUGR is already suspected [13, 14]. Unfortunately, there are inconsistencies between these guidelines. For example, the 2008 NVOG guideline only briefly mentions SFH measurements, whereas the 2013 KNOV guideline introduces it as the designated method for monitoring fetal growth [14]. Furthermore, certain aspects of clinical practice such as indications for additional testing are not specified, leaving room for personal interpretation and leading to a wide variation of practice among midwives and among obstetricians.

In the IUGR Risk Selection (IRIS) study the effectiveness and cost-effectiveness of routine third trimester ultrasound for monitoring fetal growth in comparison with usual care will be evaluated in low-risk pregnancies [24]. The outcomes of this study will be affected by the subsequent diagnostics and management of pregnancies with suspected IUGR, requesting a uniform approach by all the professionals involved. However, a lack of consistent scientific evidence leads to wide variations in some clinical practices. We therefore developed uniform multidisciplinary recommendations by carrying out a Delphi study.

The main purpose of the Delphi study reported here was to achieve consensus on issues where evidence is still lacking or where discrepancies were identified on screening for, diagnosis and management of IUGR among professionals in a multidisciplinary care setting.

## Methods

### Delphi technique

We chose a Delphi technique because this allows for consensus among panellists with different backgrounds and possibly conflicting interests, obtained through several rounds of structured questionnaires accompanied by substantive arguments provided by the panel itself. We planned three rounds and asked panellists to respond within three weeks in each round. Non-responders were reminded by personal mailing and eventually a telephone call.

Before the start of data collection we aimed for a panel of at least 50 members and we defined consensus a priori as agreement between the professional groups on the same answer and among at least 70% of the panellists within groups. The panellists were explicitly informed about this definition of consensus.

### Selection of the Delphi panel

To facilitate multidisciplinary support for the recommendations being developed in this Delphi study, both midwives and obstetricians were recruited throughout the Netherlands using email, telephone and tweets in August and September 2013. We aimed for an equal representation of midwife-led and obstetrician-led care. All potential participants were requested to complete a short questionnaire allowing us to select participants based on their professional expertise and setting. In addition, we personally approached midwives and obstetricians who were involved in the development of the Dutch guidelines for IUGR and researchers on IUGR or fetal monitoring.

Panellists remained anonymous to each other and received no financial or other compensation for their participation.

### Preparation of the Delphi questionnaires

The evidence based, monodisciplinary KNOV and NVOG guidelines were the leading sources for the development of the questionnaires as these are applied in current practice [13, 14]. Furthermore, we used the more recent guideline of the Royal College of Obstetricians and Gynaecologists (RCOG) because it incorporates screening in the general population as well as additional diagnostics and management for the population at risk for IUGR [12]. The British antenatal care also involves multidisciplinary collaboration between midwives or general practitioners in the community and obstetricians. In addition, the RCOG guideline explicitly incorporates SFH measurements plotted on a customised growth chart as recommended in the KNOV guideline [12].

**Fig. 1:**
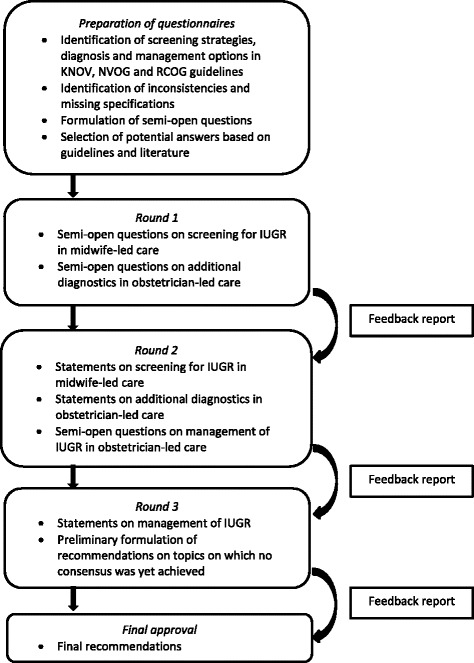
The Delphi procedure. KNOV = Royal Dutch Organisation of Midwives, NVOG = Dutch Society of Obstetrics and Gynaecology, RCOG = Royal College of Obstetricians and Gynaecologists, IUGR = intrauterine growth restriction

Screening strategies, additional diagnostics and management options were identified in these guidelines (Figure 1). We rephrased the detected inconsistencies and unspecified thresholds for diagnostic tests or interventions in questions. Potential answers were also based on these guidelines or searched for in recent literature.

The project group consisting of all authors approved the content and phrasing of the questions and the response options. For some items, additional advice was asked from an obstetrician and sonographer from the IRIS study group.

### The rounds

This Delphi study consisted of three rounds of questionnaires, submitted online between September 25th 2013 and January 2nd 2014 (Fig. 1).

The **first round** focused on screening for IUGR in midwife-led care and on diagnostic tests to be considered in obstetrician-led care if IUGR is suspected. It also incorporated questions about quality of ultrasound biometry as this is mentioned, though not specified, by the KNOV guideline [14]. For each **question**, the panellists were asked to select their preferred answer or make another suggestion under the response option “other”. In every consecutive round, the answers given in the previous round were presented in tables with both absolute numbers and percentages, categorised per professional group. The questionnaire was also accompanied by a feedback report of all explanations for every answer provided by the panellists, offering the panellists the opportunity to reconsider their opinion in relation to the answers of the complete panel.

For **example**, a question in the first round was: “How should slow growth in SFH measurements be defined?” Panellists were asked to choose between: “by eye-balling”, “with a decrease of a specified number of centiles on the customised growth chart (CGC)”, “with a combination of both previous methods”, “no opinion” or “other”. Panellists were encouraged to explain their choices.

Only if no consensus was reached, the question was rephrased in the **second round** in a **statement** based upon the given answers and comments (Fig. 1). For **example**: “Slow growth in SFH measurements should be defined with a decrease of a specified number of centiles on the CGC”, as this option was preferred most in round 1. The panellists were asked to rate their (dis)agreement on a Likert scale, including the option “no opinion” so professionals from different disciplines were not forced to decide on specific topics beyond their expertise. For the calculation of the rates of agreement, this option was excluded. **Questions** about management of IUGR in secondary or tertiary care were added to these statements in the second round.

In the **third round** we similarly asked the panellists to score **statements** for which consensus had not been reached, relating them to the recommendations for the IRIS study. In our **example**: “In the IRIS protocol we will advise to define slow growth in SFH measurements with a decrease of a specified number of centiles on the CGC. Eye-balling is of secondary importance.” No new questions were added in this round.

The multidisciplinary project group considered the importance of the statements for which still no consensus was achieved in the final round in the perspective of the IRIS study. For those considered important and possible based on the results of the Delphi procedure, the project group formulated a recommendation for the IRIS study. For all recommendations it was explicitly mentioned whether it was based on a guideline, the Delphi procedure or a decision of the project group. The Delphi panel was asked for a final approval of all the resulting recommendations before the start of the IRIS study.

## Results

In total, 84 professionals responded. From these we selected the panellists warranting an equal representation of midwife-led and obstetrician-led care, experience with SFH measurements and ultrasonography. We also warranted a sample of Dutch regions and tertiary care centres. This led to 56 panellists: 27 representing midwife-led care and 29 obstetrician-led care. Two midwives who solely worked in a hospital were assigned to the obstetrician-led care group. Their characteristics are shown in Table 1.

**Table 1:** Characteristics of the Delphi panel

Characteristic	Midwife-led Care Panellist	Obstetrician-led Care Panellist			Total group
	*N* = 27 (48%)	*N* = 29 (52%)			*N* = 56 (100%)
		Secondary care	Tertiary care	Combined	
Years of experience in current position, mean (range)	16.30 (3–39)	12.32 (1–40)			14.97 (1–40)
Midwife	11 (19.6%)				11 (19.6%)
+ sonographer	12 (21.4%)	0	1 (1.8%)	1 (1.8%)	14 (25.0%)
+ policy and guideline development	4 (7.1%)				4 (7.1%)
Obstetrician		11 (19.6%)	5 (8.9%)	1 (1.8%)	17 (30.4%)
+ perinatologist		2 (3.6%)	2 (3.6%)	1 (1.8%)	5 (8.9%)
+ policy & guideline development and perinatologist		1 (1.8%)	3 (5.4%)	0	4 (7.1%)
Expert sonographer		0	1 (1.8%)	0	1 (1.8%)
Work address					
Drenthe	0	0			0
Flevoland	0	0			0
Friesland	2 (3.6%)	0			2 (3.6%)
Gelderland	4 (7.1%)	2 (3.6%)			6 (10.7%)
Groningen	0	1 (1.8%)			1 (1.8%)
Limburg	2 (3.6%)	2 (3.6%)			4 (7.1%)
North Brabant	4 (7.1%)	3 (5.4%)			7 (12.5%)
North Holland	3 (5.4%)	11 (19.6%)			14 (25.0%)
Overijssel	1 (1.8%)	1 (1.8%)			2 (3.6%)
South Holland	4 (7.1%)	5 (8.9%)			9 (16.1%)
Utrecht	6 (10.7%)	4 (7.1%)			10 (17.9%)
Zeeland	1 (1.8%)	0			1 (1.8%)

Percentages do not always add up to 100% due to rounding error

**Fig. 2:**
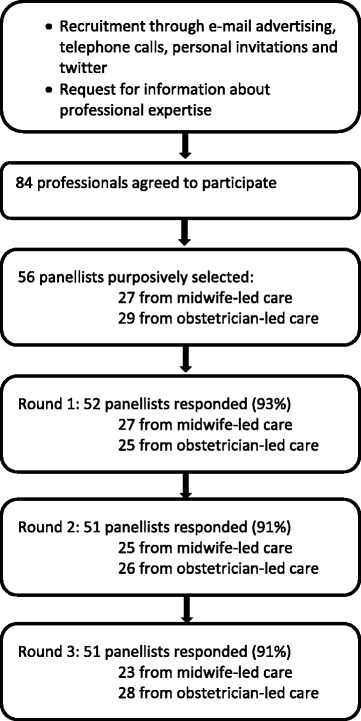
Flowchart of participation per Delphi round

Figure 2 shows the participation rate (range 91% to 93%) per professional group per round. All panellists participated in at least one round, 45 (80%) panellists participated in all three rounds, two (4%) dropped out after completing the first round in spite of several reminders. The main reason for not participating was lack of time.

### Screening

Table 2 shows the questions and statements concerning screening in midwife-led care for IUGR after 26 weeks gestational age. The Delphi procedure resulted in four consensus based recommendations and one formulated by the project group.

**Table 2:** Screening for IUGR in midwife-led care at a gestational age ≥ 26 weeks 0 days: opinion per level of care

Statement	Answer	Midwife-led Care	Obstetrician-led Care	Consensus total group
		*n* (%)	n (%)	
1.1. Slow growth should be defined as a decrease of a specified number of centiles of SFH measurements on the CGC. Eye-balling is of secondary importance.	A	20 (87%)	26 (93%)	**Consensus: agree**
	D	3 (13%)	2 (7%)	
	N	0	0	
	M	4	1	
1.2. Slow growth is a decrease of at least 20 centiles *(*e.g. *from P70 to P50, with a minimum interval of 2 weeks)* of SFH measurements on the CGC. This is an indication for an ultrasound biometry.	A	20 (91%)	20 (91%)	**Consensus: agree**
	D	2 (9%)	2 (9%)	
	N	3	4	
	M	2	3	
1.3. With ultrasound biometry, slow growth should be stated as a decrease of a specified number of centiles of EFW on the CGC. Eye-balling is of secondary importance in this evaluation.	A	22 (96%)	25 (89%)	**Consensus: agree**
	D	1 (4%)	3 (11%)	
	N	0	0	
	M	4	1	
1.4. With ultrasound biometry, slow growth is a decrease of at least 20 centiles *(*e.g. *from P70 to P50, with a minimum interval of 2 weeks)* of EFW on the CGC. This is an indication for referral to obstetrician-led care.	A	20 (91%)	20 (83%)	**Consensus: agree**
	D	2 (9%)	4 (17%)	
	N	2	2	
	M	3	3	
1.5. In the IRIS study it will be advised, not obligatory, that two consecutive biometry ultrasounds are performed by the same sonographer	A	22 (96%)	20 (71%)	**Consensus: agree**
	D	1 (4%)	8 (29%)	
	N	0	0	
	M	4	1	
1.6. To guarantee quality in the IRIS study, sonographers who are trained for the 18–23 weeks FAS are preferable, however other sonographers are acceptable if at least trained in biometry until 3rd trimester.	A	18 (82%)	23 (82%)	**Consensus: agree**
	D	4 (18%)	5 (18%)	
	N	1	0	
	M	4	1	
1.7. To guarantee quality, sonographers should obtain a minimum number of credits from their professional organization, by following a training once a year.	A	20 (83%)	17 (71%)	**Consensus: agree**
	D	4 (17%)	7 (29%)	
	N	0	2	
	M	3	3	
1.8. To guarantee quality, sonographers should perform at least 100 biometry ultrasounds a year	A	18 (90%)	22 (96%)	**Consensus: agree**
	D	2 (10%)	1 (4%)	
	N	4	3	
	M	3	3	
1.9. Ultrasound quality should be checked yearly, evaluation of a log should be an essential part of this	A	20 (87%)	19 (86%)	**Consensus: agree**
	D	3 (13%)	3 (14%)	
	N	1	4	
	M	3	3	
1.10. The ultrasound machine should meet the requirements for 18–23 weeks FAS as stated by the NVOG quality norm ‘Fetal ultrasound’^16^	A	12 (80%)	15 (79%)	**Consensus: agree**
	D	3 (20%)	4 (21%)	
	N	9	7	
	M	3	3	
1.11. Which cut-off value for the single deepest vertical pocket measurement for assessing amniotic fluid volume is an indication for referral to obstetrician-led care?	< P 2.3	0	2 (7%)	No consensus
	< P5	6 (40%)	11 (39%)	
	<2 cm (regardless of gestational age)	9 (60%)	15 (54%)	
	N	8	0	
	M	4	1	

*A* agree, *D* disagree, *N* no opinion/expertise, *M* missing: panellist has not participated in this round or has not answered this question, **Consensus** = ≥70% of panellists per level of care agree and both groups agree upon the same. Percentages do not always add up to 100% due to rounding error

*IUGR* intrauterine growth restriction, *SFH* serial fundal height, *CGC* customised growth chart, *EFW* estimated fetal weight, *IRIS* IUGR risk selection, *FAS* fetal anomaly scan, *NVOG* Dutch Society of Obstetrics and Gynaecology

After three rounds, the majority of professionals agreed that slow growth should be defined by a decrease of a specified number of centiles, both for SFH measurements as for ultrasound biometry, rather than through eye-balling alone (statements 1.1 and 1.3). In the first round, panellists who considered the specification of a number of centiles necessary for defining slow growth in SFH measurements, suggested to use at least 2, 10 or 20 centiles as cut-off points. In the second round, consensus was achieved about using a minimum decrease of 20 centiles; no other options had been added by the panellists (statement 1.2).

For EFW based on ultrasound, a decrease of at least 15 or 20 centiles was suggested in the first round, leading to consensus in the second round on a decrease of 20 centiles as the appropriate threshold for referral to obstetrician-led care (statement 1.4). The panel suggested that the same sonographer should perform the consecutive biometry ultrasounds (statement 1.5).

Although there is no compulsory audit for ultrasound biometry in the Netherlands, the majority of panellists agreed upon several quality norms as shown in Table 2. For the sonographers the following consensus was reached: being trained for the 18–23 weeks fetal anomaly scan, repeated education by participating in a training at least once a year, performance of at least 100 biometry scans a year and the yearly evaluation of a log (describing all exceptional findings) (statements 1.6-1.9). For the ultrasound machine it was agreed that it should meet the high standard as stated for the 18–23 weeks fetal anomaly scan [25] (statement 1.10). No consensus was achieved for the specification of the cut-off value for decreased amniotic fluid volume (statement 1.11). The project group made the recommendation to refer pregnant women to obstetrician-led care if the single deepest vertical pocket is below 2 cm.

### Diagnosis

Table 3 presents the questions and statements about additional diagnostics in obstetrician-led care after referral for IUGR after 26 weeks gestational age. The Delphi technique has led to eight consensus based recommendations and one formulated by the project group.

**Table 3:** Additional diagnostic tests in case of suspected IUGR in obstetrician-led care after referral from midwife-led care at a gestational age ≥ 26 weeks 0 days: opinion per level of care

Statement	Answer	Midwife-led Care	Obstetrician-led Care	Consensus total group
		n (%)	n (%)	
2.1. An ultrasound biometry and assessment of amniotic fluid volume is to be repeated immediately after referral to obstetrician-led care, even if this is within 2 weeks of the previous scan (in midwife-led care).	A	12 (60%)	20 (80%)	No consensus
	D	8 (40%)	5 (20%)	
	N	3	1	
	M	4	3	
2.2. As fetal growth can only be evaluated through serial measurements, we will advise to plot EFW on the CGC in obstetrician-led care as well (as in midwife-led care). We will also advise to be alert for asymmetrical growth based on the ratios of AC, FL, BPD and HC.	A	23 (100%)	20 (80%)	**Consensus: agree**
	D	0	5 (20%)	
	N	0	2	
	M	4	2	
2.3. In obstetrician-led care, decreased amniotic fluid volume should be defined using the same cut-off values as in midwife-led care.	A	23 (100%)	24 (96%)	**Consensus: agree**
	D	0	1 (4%)	
	N	0	1	
	M	4	3	
2.4. Suspicion of IUGR is an indication for measuring the umbilical artery Doppler in obstetrician-led care. Which measurement is the first abnormal sign for fetal deterioration? *(multiple options)*	Pulsatility Index (PI)	10 (91%)	23 (92%)	**Consensus: Pulsatility Index (PI)** No consensus on the other answers
	Resistance Index (RI)	4 (36%)	0	
	Absent diastolic flow	2 (18%)	20 (80%)	
	Reversed diastolic flow	3 (27%)	18 (72%)	
	Other	0	0	
	N	16	0	
	M	0	4	
2.5. A PI of the umbilical artery Doppler ≥ P95 is abnormal (and management of pregnancy should be adjusted).	A	11 (92%)	24 (96%)	**Consensus: agree**
	D	1 (8%)	1 (4%)	
	N	11	1	
	M	4	3	
2.6. In the IRIS study it will be advised to assess the PI of the middle cerebral artery Doppler when IUGR is suspected.	A	10 (100%)	21 (88%)	**Consensus: agree**
	D	0	3 (12%)	
	N	13	3	
	M	4	2	
2.7. The ductus venosus Doppler should be measured when IUGR is suspected.	A	5 (83%)	6 (46%)	No consensus
	D	1 (17%)	7 (54%)	
	N	17	13	
	M	4	3	
2.8. In the IRIS study we will recommend a FAS in case of IUGR, if not previously performed.	A	22 (100%)	24 (89%)	**Consensus: agree**
	D	0	3 (11%)	
	N	1	0	
	M	4	2	
2.9. At which degree of IUGR, defined by centiles of EFW on the CGC, should a FAS be offered to the pregnant woman?	≤ P5	12 (60%)	12 (50%)	No consensus
	≤ P2.3	7 (35%)	10 (42%)	
	Degree of IUGR is not relevant for the assessment of fetal anatomical anomalies	1 (5%)	2 (8%)	
	N	3	3	
	M	4	2	
2.10. A FAS because of suspected IUGR, should be performed by:	A sonographer in secondary care, who is trained for FAS. Depending on the results, referral for advanced sonography in tertiary care will take place	11 (55%)	6 (22%)	No consensus
	An obstetric ultrasound specialist, trained for advanced sonography (under the responsibility of tertiary care)	9 (45%)	21 (78%)	
	N	3	0	
	M	4	2	
2.11. In the IRIS study, in case of suspicion of IUGR, it will be advised not to commence CTG monitoring as long as there is no decrease in fetal movements nor a hypertensive disorder and no abnormal Doppler profiles.	A	18 (90%)	23 (85%)	**Consensus: agree**
	D	2 (10%)	4 (15%)	
	N	3	0	
	M	4	2	
2.12. At which degree of IUGR, defined by centiles of EFW on the CGC, should assessment for specific fetal infections be advised?	< P10	2 (12%)	1 (4%)	No consensus
	< P5	5 (29%)	7 (27%)	
	< P2.3	10 (59%)	16 (61%)	
	At another *P*-value	0	2 (8%)	
	N	6	1	
	M	4	2	
2.13. Gestational age, in addition to degree of IUGR, determines whether one should check for specific fetal infections	A	2 (12%)	4 (16%)	**Consensus: disagree**
	D	15 (88%)	21 (84%)	
	N	8	1	
	M	2	3	
2.14. If fetal infections are to be checked for because of suspicion of IUGR than test for: Coxsackie Virus	A	3 (14%)	0	**Consensus: disagree**
	D	18 (86%)	23 (100%)	
	N	6	2	
	M	0	4	
Cytomegalovirus	A	19 (90%)	20 (87%)	**Consensus: agree**
	D	2 (10%)	3 (13%)	
	N	6	2	
	M	0	4	
Malaria	A	1 (5%)	0	**Consensus: disagree**
	D	20 (95%)	23 (100%)	
	N	6	2	
	M	0	4	
Toxoplasmosis	A	17 (100%)	19 (76%)	**Consensus: agree**
	D	0	6 (24%)	
	N	6	1	
	M	4	3	
Rubella	A	14 (88%)	11 (55%)	No consensus
	D	2 (12%)	9 (45%)	
	N	7	6	
	M	4	3	
Herpes	A	13 (100%)	12 (55%)	No consensus
	D	0	10 (45%)	
	N	10	4	
	M	4	3	
Parvo B19	A	18 (100%)	14 (70%)	**Consensus: agree**
	D	0	6 (30%)	
	N	5	6	
	M	4	3	
Syphilis	A	11 (79%)	8 (40%)	No consensus
	D	3 (21%)	12 (60%)	
	N	9	6	
	M	4	3	
2.15. In the IRIS study, offering invasive prenatal testing will not be advised routinely in case of IUGR; but rather individual risk factors and gestational age should be considered.	A	22 (100%)	25 (93%)	**Consensus: agree**
	D	0	2 (7%)	
	N	1	0	
	M	4	2	
2.16. In the IRIS study, invasive prenatal testing will be offered to the couple if the EFW ≤ P2.3.	A	18 (95%)	23 (85%)	**Consensus: agree**
	D	1 (5%)	4 (15%)	
	N	4	0	
	M	4	2	

*A* agree, *D* disagree, *N* no opinion/expertise, *M* missing: panellist has not participated in this round or has not answered this question, **Consensus** = ≥70% of panellists per level of care agree and both groups agree upon the same. Percentages do not always add up to 100% due to rounding error

*IUGR* intrauterine growth restriction, *EFW* estimated fetal weight, *CGC* customised growth chart, *AC* abdominal circumference, *FL* femur length, *BPD* biparietal diameter, *HC* head circumference, *PI* Pulsatility Index, *IRIS* IUGR risk selection, *FAS* fetal anomaly scan, *CTG* cardiotocography

In the first round several panellists suggested to use the same threshold for decreased amniotic fluid volume for referral to secondary care as for a change in management of pregnancy if already referred. This was rephrased in a statement, reaching consensus in the second round (statement 2.3).

The panellists agreed that when IUGR is suspected at a gestational age of at least 26 weeks, a pulsatility index (PI) of the umbilical artery Doppler of at least the 95th centile would be a first sign for the placental blood supply not meeting the fetal demand, necessitating a change in monitoring and/or management (statements 2.4-2.5). This consensus was achieved after considering measuring the resistance index, additional checking for absent or reversed diastolic flow and other options. Consecutively, based on the answers, the 90th and 95th centile of the PI have been considered as thresholds for changing the monitoring or management of pregnancy. In addition to the umbilical artery Doppler, panellists agreed on the measurement of the PI of the middle cerebral artery Doppler (statement 2.6). No consensus was achieved on the measurement of the ductus venosus Doppler in this case (statement 2.7). Some panellists explained that results of the Trial of Umbilical and Fetal Flow in Europe were soon to be expected and therefore should be waited for to decide about the ductus venosus Doppler [26]. The project group complied with this: no recommendation was made.

For pregnant women without a fetal anomaly scan, the panellists agreed to recommend one if IUGR is suspected (statement 2.8). However, agreement about the indication specified in the degree of IUGR; or who should perform this ultrasound was not achieved (statements 2.9-2.10). The project group has recommended to offer an advanced anomaly scan starting from an EFW of P2.3 or below.

Consensus was achieved that cardiotocography (CTG) monitoring is not indicated in case of suspicion of IUGR as long as there is no decrease in fetal movements, nor a hypertensive disorder and no abnormal Dopplers (statement 2.11).

Gestational age was not regarded an important factor in deciding to check for infections (statement 2.13). Although the degree of IUGR was considered relevant to this decision, no consensus was reached on the threshold for the EFW centile (statement 2.12). Therefore the project group has recommended an EFW at P2.3 or below as the appropriate cut-off point. Toxoplasmosis, Cytomegalovirus and Parvo B19 are infections to be tested for, but not Coxsackie Virus and Malaria. The panel did not reach consensus upon testing for Rubella, Herpes and Syphilis (statement 2.14).

### Management

Questions and statements about further management of IUGR pregnancies in obstetrician-led care are shown in Table 4. The Delphi procedure has led to eight consensus based recommendations and two formulated by the project group. Consensus was reached that if the EFW is below the fifth centile, the pregnant woman should remain in obstetrician-led care with ultrasound biometry repeated every two weeks (statement 3.1 and 3.3). Disagreement remained whether a pregnancy with an EFW between the fifth and tenth centile and no abnormal results from additional testing, should be monitored in midwife-led care with serial ultrasounds or in obstetrician-led care (statement 3.2). No further recommendation was made by the project group leaving the decision up to the individual professional involved. Additional monitoring of IUGR by the assessment of the amniotic fluid volume and Doppler velocimetry was agreed, however no consensus was achieved on its frequency (statements 3.4- 3.5). The project group advised a repetition of at least every two weeks in combination with the biometry.

**Table 4:** Management in obstetrician-led care in case of suspected IUGR at a gestational age ≥ 26 weeks 0 days: opinion per level of care

Statement	Answer	Midwife-led Care	Obstetrician-led Care	Consensus total group
		n (%)	n (%)	
3.1. If additional tests (Doppler, amniotic fluid volume, FAS and on indication: invasive prenatal testing and assessment of infections) show no anomalies, in which level of care should the pregnancy with EFW (and/or AC) P2.3 - P5 be continued?	Continue in midwife-led care	0	0	**Consensus: Continue in obstetrician-led care**
	Continue in midwife-led care and offer serial ultrasound biometry	6 (26%)	3 (12%)	
	Continue in obstetrician-led care	17 (74%)	23 (88%)	
	N	0	0	
	M	4	3	
3.2. If the EFW on the CGC is P5-P10, the pregnancy should be continued:	In midwife-led care with serial ultrasound biometry	18 (78%)	12 (44%)	No consensus
	In obstetrician-led care	5 (22%)	15 (56%)	
	N	0	0	
	M	4	2	
3.3. If a pregnancy needs to be monitored in obstetrician-led care because of suspicion of IUGR, ultrasound biometry should be repeated:	Every day	0	0	**Consensus: Every 2 weeks**
	Every other day	0	0	
	Twice a week	0	2 (8%)	
	Once a week	3 (16%)	2 (8%)	
	Every 10 days	1 (5%)	2 (8%)	
	Every 2 weeks	14 (74%)	18 (72%)	
	With another frequency	1 (5%)	1 (4%)	
	Ultrasound biometry should not be part of the routine monitoring	0	0	
	N	4	1	
	M	4	3	
3.4. If a pregnancy needs to be monitored in obstetrician-led care because of suspicion of IUGR, assessment of the amniotic fluid volume should be repeated:	Every day	0	0	No consensus
	Every other day	0	0	
	Twice a week	1 (5%)	1 (4%)	
	Once a week	4 (21%)	18 (72%)	
	Every 10 days	1 (5%)	1 (4%)	
	Every 2 weeks	9 (48%)	2 (8%)	
	With another frequency	3 (16%)	3 (12%)	
	Amniotic fluid volume should not be routinely monitored	1 (5%)	0	
	N	4	1	
	M	4	3	
3.5. If a pregnancy needs to be monitored in obstetrician-led care because of suspicion of IUGR, assessment of the umbilical artery Doppler should be repeated:	Every day	0	0	No consensus
	Every other day	0	0	
	Twice a week	2 (11%)	2 (8%)	
	Once a week	7 (39%)	15 (60%)	
	Every 10 days	1 (5%)	1 (4%)	
	Every 2 weeks	2 (11%)	0	
	With another frequency	5 (28%)	7 (28%)	
	Umbilical artery Doppler should not be routinely monitored	1 (5%)	0	
	N	5	1	
	M	4	3	
3.6. In the IRIS study a tertiary care centre should be consulted about the administration of MgSO4 for fetal neuroprotection if there is suspicion of severe IUGR at a gestational age < 32 weeks 0 days.	A	6 (100%)	20 (87%)	**Consensus: agree**
	D	0	3 (13%)	
	N	17	4	
	M	4	2	

*A* agree, *D* disagree, *N* no opinion/expertise, *M* missing: panellist has not participated in this round or has not answered this question, **Consensus** = ≥70% of panellists per level of care agree and both groups agree upon the same. Percentages do not always add up to 100% due to rounding error

*IUGR* intrauterine growth restriction, *FAS* fetal anomaly scan, *EFW* estimated fetal weight, *AC* abdominal circumference, *CGC* customised growth chart, *IRIS* IUGR risk selection, *MgSO4* magnesium sulphate

Because of the variation in responses on when to refer to tertiary care, we did not reach the stage of formulating statements. The project group has decided to recommend that if there is a reasonable chance of a (necessary) birth before 32 weeks of gestation and/or if the EFW is below 1250 g, the woman should be referred to tertiary care, in accordance to the NVOG guideline.

Also the questions about when induction of labour or a primary caesarean section was indicated never reached the stage of formulating statements, even though the statements concerned IUGR without further abnormalities in additional diagnostics or monitoring. In the third round we tried to narrow it down by suggesting to combine gestational age with degree of IUGR defined as a centile for EFW and/or abdominal circumference. Nevertheless, no uniformity could be detected in the given answers regardless of professional group, refraining the project group from formulating a recommendation.

## Discussion

### Summary of the main findings

In this Delphi study consensus was achieved between professionals working in midwife-led and obstetrician-led care on 27 statements, resulting in twenty recommendations for prenatal care for IUGR; this may contribute to optimizing the multidisciplinary teamwork throughout all levels of perinatal care. Consensus was not reached on some statements that were important for the IRIS study, therefore the multidisciplinary project group formulated four recommendations. No recommendations could be made about when to induce labour in absence of further abnormalities in additional diagnostics or monitoring, nor about when to perform a primary caesarean section.

### Comparison with other studies or literature

Consensus was reached on the definition of slow growth as a decrease of 20 centiles in both SFH and EFW measurements plotted on the CGC with a minimum interval of two weeks. As far as we know, this is the first specification of slow growth reported in the literature. Guidelines that mention slow growth do not define it or only generally describe it as a crossing of centiles in a downward direction [10–12, 14]. The Fetal Growth Longitudinal Study of the Intergrowth-21st Project has recommended international growth standards based on fetal ultrasound measurements: head and abdominal circumference, biparietal diameter, occipitofrontal diameter and femur length [21]. However, they do not define slow growth [21]. Further research is needed to investigate whether this criterion for slow growth is associated with an increased risk of adverse perinatal outcome.

The panellists achieved consensus on quality norms for fetal biometry ultrasounds. Multiple national and international societies have established guidelines and audit systems for assessing the quality of the nuchal translucency scan or the 18–23 weeks fetal anomaly scan [25, 27, 28]. Although recommended in the literature, no audit system for the quality of biometry exists on a national or international level [29–33]. This is surprising considering the role of biometry in diagnosing and managing IUGR [1–5]. In this Delphi study consensus was reached for requirements for both the ultrasound machine and the sonographer. This could offer a basis for the development of quality audits and certification in biometry. Further research is needed to consider these requirements from an educational or quality assessment perspective, as the panellists were primarily selected for their expertise on IUGR rather than on education or quality assessment.

Doppler velocimetry can be used to monitor redistribution patterns of blood flow in the pregnancy with IUGR, suggesting a risk for further fetal compromise [15, 17, 34]. However, besides assessment of the umbilical artery in the third trimester of pregnancy, Doppler measurements of various other vessels are suggested in guidelines, without specification of thresholds [10–13, 35]. In this Delphi study the panel specified abnormal flow as an umbilical artery PI of 95% and above. In addition, the measurement of the middle cerebral artery Doppler PI was considered to contribute to the surveillance in case of IUGR, but the threshold for abnormal flow was not specified.

Unlike the American Congress of Obstetricians and Gynecologists (ACOG) guideline, but in line with the RCOG and the Society of Obstetricians and Gynaecologists of Canada (SOGC) guideline, the panel agreed not to advise CTG monitoring for IUGR, as long as other measurements such as Doppler velocimetry remain normal and without a decrease of fetal movements or hypertensive disorders [10–12].

No recommendations were made about induction of labour versus expectant monitoring based on the combination of gestational age and degree of IUGR in absence of further abnormalities in additional tests. The panellists, regardless of their professional group, indicated various things and thresholds to consider for each pregnancy individually. The RCOG guideline recommends a senior obstetrician to determine the timing and mode of birth in pregnancies with a small-for-gestational-age fetus detected after 32 weeks with normal umbilical artery Doppler. Furthermore they recommend that delivery should be offered at 37 weeks of gestation [12]. In the Disproportionate Intrauterine Growth Intervention Trial At Term, no important differences in adverse outcomes were found between both strategies [36, 37]. The project group also left the decision up to the obstetrician involved as support for a general recommendation was lacking.

### Strengths and limitations

A strength of the study is that we could select a panel of 56 members from 84 candidates, equally representing professionals from midwife-led and obstetrician-led care from a wide range of the Dutch geographical areas. Although there is no uniform recommendation about the size of a Delphi panel, we exceeded our predefined number of 50 which seems fair compared to other studies [38]. We deem expertise on IUGR to be properly represented in this Delphi panel, therefore, content validity of the results may be assumed [39]. The response rate of 91-93% of panellists per round was high, exceeding the recommendation of 70% per round suggested by others [40, 41].

Implementation of and adherence to guidelines for clinical practice is known to be complex [42–44]. Through the active involvement of this multidisciplinary panel from all over the country we aim to improve bottom up support for the recommendations in the IRIS study. This may lead to perceptions of ownership and acceptance of more uniform recommendations, which in turn might increase implementation in daily practice [43, 45].

While there is no general rule about how consensus should be defined in a Delphi study, levels starting from 51% agreement have been described [38, 46]. Compared to this our criterion stated a priori of at least 70% seems reasonable and suggests enough support in daily practice for the emerging recommendations. Furthermore, this percentage is a means to stimulate achieving consensus among the majority of professionals involved rather than a goal in itself. As we did not count the answer “no opinion” for the rate of agreement, it was possible to effectively use the panellists with expertise on the concerning topic. Panellists often explained they had no expertise concerning the particular subject if they chose this option. For example, a primary care midwife was not obliged to (dis)agree about when magnesium sulphate should be administered, as this is no part of her training nor her discipline.

## Conclusions

In this study we achieved consensus within a multidisciplinary panel on 27 statements about prenatal care for IUGR. This has led to twenty consensus based recommendations that will be implemented in a study on the effectiveness and cost-effectiveness of routine third trimester ultrasound for monitoring fetal growth.

## Abbreviations

AC: Abdominal circumference
BPD: Biparietal diameter
CGC: Customised growth chart
CTG: Cardiotocography
EFW: Estimated fetal weight
FAS: Fetal anomaly scan
FL: Femur length
HC: Head circumference
IRIS study: IUGR risk selection study
IUGR: Intrauterine growth restriction
KNOV: Royal Dutch Organisation of Midwives
MgSO4: Magnesium sulphate
NVOG: Dutch Society of Obstetrics and Gynaecology
PI: Pulsatility Index
RCOG: Royal College of Obstetricians and Gynaecologists
SFH: Serial fundal height
